# Detection and Quantification of *Stagonosporopsis cucurbitacearum* in Seeds of *Cucurbita maxima* Using Droplet Digital Polymerase Chain Reaction

**DOI:** 10.3389/fmicb.2021.764447

**Published:** 2022-01-11

**Authors:** Sergio Murolo, Marwa Moumni, Valeria Mancini, Mohamed Bechir Allagui, Lucia Landi, Gianfranco Romanazzi

**Affiliations:** ^1^Department of Agricultural, Food and Environmental Sciences, Marche Polytechnic University, Ancona, Italy; ^2^Laboratory of Plant Protection, National Institute for Agronomic Research of Tunisia, University of Carthage, Ariana, Tunisia

**Keywords:** etiology, fungal pathogens, molecular, pathogen detection, techniques

## Abstract

*Stagonosporopsis cucurbitacearum* is an important seedborne pathogen of squash (*Cucurbita maxima*). The aim of our work was to develop a rapid and sensitive diagnostic tool for detection and quantification of *S. cucurbitacearum* in squash seed samples, to be compared with blotter analysis, that is the current official seed test. In blotter analysis, 29 of 31 seed samples were identified as infected, with contamination from 1.5 to 65.4%. A new set of primers (DB1F/R) was validated *in silico* and in conventional, quantitative real-time PCR (qPCR) and droplet digital (dd) PCR. The limit of detection of *S. cucurbitacearum* DNA for conventional PCR was ∼1.82 × 10^–2^ ng, with 17 of 19 seed samples positive. The limit of detection for ddPCR was 3.6 × 10^–3^ ng, which corresponded to 0.2 copies/μl. Detection carried out with artificial samples revealed no interference in the absolute quantification when the seed samples were diluted to 20 ng. All seed samples that showed *S. cucurbitacearum* contamination in the blotter analysis were highly correlated with the absolute quantification of *S. cucurbitacearum* DNA (copies/μl) in ddPCR (*R*^2^ = 0.986; *p* ≤ 0.01). Our ddPCR protocol provided rapid detection and absolute quantification of *S. cucurbitacearum*, offering a useful support to the standard procedure.

## Introduction

Pumpkin, squash, and gourds (*Cucurbita* spp.) are grown throughout the world with a total production of 22.9 million tonnes, which 20.9% is produced in Europe in 2019 (FAOSTAT, 2021). Italy is ranked 9th in the world with 569.120 tonnes in terms of total production (FAOSTAT, 2021).

Several diseases have economic impacts on the production of the *Cucurbita* spp. (squash; cucurbit). These include gummy stem blight (GSB) (with foliar symptoms) and black rot (with fruit symptoms), which are caused by three species of the fungi *Stagonosporopsis*: (i) *Stagonosporopsis cucurbitacearum* (Fr.) Aveskamp, Gruyter, and Verkley [anamorph *Phoma cucurbitacearum* (Fr.) Sacc.], synonym *Didymella bryoniae* (Fuckel) Rehm; (ii) *Stagonosporopsis caricae* (Syd. and P. Syd.) Aveskamp, Gruyter, and Verkley (synonym *Mycosphaerella caricae* Syd. and P. Syd.); and (iii) *Stagonosporopsis citrulli* M. T. Brewer and J. E. Stewart (Stewart et al., 2015).

In the Mediterranean areas as well in Asia, *S. cucurbitacearum* was described as the main pathogenic fungus of *Cucurbita maxima* seedling related to GSB, able to reduce both squash yield and quality (Moumni et al., 2019, 2020; Zhao et al., 2020). In warm and humid environments, infections of these fungi can result 15 to 50% in reduction of yields and rapid death of the cucurbit plants (Keinath et al., 1995; Boughalleb et al., 2007; Keinath, 2011; Li et al., 2015; Yao et al., 2016; Moumni et al., 2019; Zitter and Kyle, 1992). These diseases are known to spread in the greenhouse during the growth season through airborne ascospores and by conidia transported in water on the plant surfaces, with further spread by contact between plants, or between plants and man, and onto the host plant (De Neergaard, 1989; Keinath, 1996, 2011). Thus, under favorable environmental conditions, a low level of latently infected seedlings can potentially result in a major disease epidemic (Ling et al., 2010). Moreover, *S. cucurbitacearum* is both an external and internal seedborne pathogen, which can thus carry inoculum from the seeds to the plants, although it is found mainly on the seed coat (Sudisha et al., 2006).

For these reasons, the production and the movement of seeds represent a particularly efficient vehicle to disperse such seedborne pathogens (Zhang et al., 2018; Moumni et al., 2020; Zhao et al., 2020). Also, as global trade has increased, so has the movement of seeds and other plant materials between countries, thus increasing the risk of the transport and transfer of plant pathogens (Epanchin-Niell and Hastings, 2010; Mancini and Romanazzi, 2014; Mancini et al., 2016). The introduction of exotic pathogens in this way can have catastrophic effects on both natural and agricultural ecosystems, and can result in large economic losses from lost ecosystem services and reduced crop yields (Atallah et al., 2010; Short et al., 2015; Cunniffe et al., 2016; Epanchin-Niell, 2017). Many countries have formulated legislation to limit or prevent the introduction of exotic pathogens into new areas, and these are generally supported by detection techniques (Choudhury et al., 2017). However, the low inoculum levels and the varied distribution of a pathogen within seed lots make the testing of seeds a difficult task.

Moumni et al. (2020) reported that the selection of healthy fruit is not sufficient to reduce the infection by this seedborne pathogen. On the other hand, cultural practices and fungicide application can have important roles in GSB management, although this pathogen has developed resistance to many fungicides that were previously very effective (Finger et al., 2014; Gimode et al., 2020; Newark et al., 2020). Therefore, early diagnosis of this pathogen is a fundamental step in the management of these crop diseases (Ora et al., 2011).

The most common methods currently used for rapid detection of agent related to GSB are based on the use of molecular detection tools. These include conventional polymerase chain reaction (PCR), PCR–enzyme-linked immunosorbent assays (PCR-ELISA) (Somai et al., 2002; Keinath et al., 2003), and quantitative real-time PCR (qPCR) (Somai et al., 2002; Ha et al., 2009; Ling et al., 2010). Recently, loop-mediated isothermal amplification assays have also been designed to detect *S. cucurbitacearum* in cucurbit seeds (Tian et al., 2016) and for infections in young muskmelon leaves with suspected early symptoms of GSB (Yao et al., 2016).

Droplet digital PCR (ddPCR) is a recent technology that provides both detection and quantification of DNA targets. Unlike other methods, ddPCR does not require standard curves of known concentrations for quantification (Huggett and Whale, 2013). Additionally, ddPCR has been shown to be more sensitive for the detection of low level of inoculum or unevenly distribution of pathogens in infected plants (Dreo et al., 2014; Selvaraj et al., 2018; del Pilar Martínez-Diz et al., 2020). ddPCR also shows high resistance to inhibitors compared to qPCR (Rački et al., 2014). Thus, ddPCR represents an ideal choice for infection testing of nursery propagation materials and seeds (Dingle et al., 2013; Rani et al., 2019).

Droplet digital PCR has been used to identify soil and seedborne pathogens, aiding plant disease diagnosis (Selvaraj et al., 2018; Liu et al., 2020; Lu et al., 2020; Yu et al., 2020).

Considering the emerging importance for squash seeds of *S. cucurbitacearum* the focus of our research, we set up a rapid and sensitive protocol, based on conventional PCR, validated also in qPCR and ddPCR. Such systems would speed up the diagnosis of GSB infection, which to date has generally been carried out using the blotter test, the official diagnostic method and requires several weeks before the results can be obtained.

## Materials and Methods

### Dot Blot Analysis and Morphological Identification

Thirty-one seed samples of squash (*Cucurbita maxima* Duchesne, cv. Bjaoui) were collected from the same number of fruits showing clear symptoms, mild symptoms of GBS, and asymptomatic samples. The fruit samples were collected in nine fields in the northwest of Tunisia in October 2015, 2016, and 2017. Each seed sample was analyzed to verify the presence of *S. cucurbitacearum* using the standard blotter method of the International Seed Testing Association (Mathur and Kongsdal, 2003). Two hundred seeds per sample were soaked for 5 min in 1% sodium hypochlorite solution, and then triple-rinsed with sterile distilled water. The seeds were dried for 2 min on sterile paper towels under a laminar flow hood. They were then placed in Petri dishes (diameter, 110 mm) on eight overlapping sterile filter paper layers (Whatman N° 4) that were moistened with 5 ml sterile distilled water, and incubated at 25°C under 12 h/12 h day/night artificial light cycles (Master TL-D Super 80 58W/830).

For each sample of seeds, 20 Petri dishes were used, each of which contained 10 seeds. From days 7 to 15 after plating, the Petri dishes were examined daily under a stereomicroscope for the presence of *S. cucurbitacearum* fungal structures. The pycnidia on the seeds were excised and morphological identification was carried out under a compound microscope. For each sample, the proportion (%) of the seeds infected by *S. cucurbitacearum* was calculated. Statistical analyses were performed using the software SPSS (version 20; IBM, Armonk, NY, United States). The data were first tested for normality and homogeneity of variance by Levene’s test. Welch’s ANOVA was performed to determine any differences in seed samples, and means were separated using the Games-Howell *post hoc* test (*P* < 0.05).

The fungal structures on the seeds that were morphologically identified as *S. cucurbitacearum* were transferred onto potato dextrose agar (Liofilchem Srl, Roseto degli Abruzzi, Italy) in Petri dishes. After 10 days on the potato dextrose agar at 23 ± 2°C, morphological identification was carried out according to the color and shape of the colonies, combined with the characteristics of the pycnidia and spores.

### DNA Extraction and Molecular Characterization of Natural Seed Inoculum

Total DNA was initially extracted from the mycelia of isolate D33 that was previously identified as *S. cucurbitacearum* by morphological and internal transcribed spacer sequence analysis (Moumni et al., 2019, 2020). In order to have a preliminary molecular characterization of the natural inoculum, the colonies, picked up from blotter test, grown in PDA and identified by morphological characters, were subjected to DNA extraction, and analyzed by a multiplex PCR able to distinguish the three morphologically similar species (*S. cucurbitacearum*, *S. citrulli*, and *S. caricae*) (Brewer et al., 2015).

For the seeds, according to the results from the blotter analysis and molecular characterization of the natural inoculum, the following were selected for further analysis: 17 seed samples as representative of *S. cucurbitacearum* infection; 2 seed samples (T18 and T101) that appeared not to be infected using the conventional method; and 1 seed sample certified as healthy (IHS) (Seminis, Monsanto Agricoltura, Italy). From each of 19 samples, 100 seeds/sample were pulverized in liquid nitrogen, with DNA extraction carried out starting from 300 mg seed tissue homogenized in 5 ml extraction buffer (3% cetyl trimethylammonium bromide, 100 mM Tris-HCl, 1.4 M NaCl, 20 mM EDTA, 2% polyvinyl pyrrolidone, and 2% sodium metabisulfite) in tissue extraction bags (12 × 15 cm; Bioreba, Switzerland). The lysates were washed with phenol/chloroform (1:1), and then chloroform. The total nucleic acids were precipitated in 1 vol. cold isopropanol, and immediately centrifuged at 13,000 × *g* for 25 min. The pellets were dried at room temperature, and then resuspended in 60 μl sterile water. The quality and quantity of the extracted DNA were evaluated using a biophotometer (Eppendorf, Hamburg, Germany).

### Validation of *Stagonosporopsis cucurbitacearum* Identification in Conventional Polymerase Chain Reaction and Sequencing

In a previous study based on random amplification of polymorphic DNA markers, Ling et al. (2010) developed primers based on sequence-characterized amplified regions (i.e., the DB17 primer set) with broad-spectrum specificity for *S. cucurbitacearum*.

Here, the two 559 bp nucleotide sequences from *S. cucurbitacearum* that are representative of the RGI and RGII molecular types (GenBank accession Nos. GQ872461 and GQ872462) were downloaded in the FASTA format and aligned using ClustalX (version 1.83) (Thompson et al., 1994). Based on the conserved sequence region common to both of these genotypes (i.e., RGI and RGII), we designed a new set of primers using the Primer3 Plus software, defined here as DBF1 (5′-TCGAATGGCTCAGAGAAGGT-3′) and DBR1 (5′-AAGTCCACGTCAGACCCATC-3′), which were then synthesized (Sigma Aldrich Merck, Darmstadt, Germany). The primers were first validated *in silico* using NCBI Primer-Blast (Ye et al., 2012). The specificity of the primers was then confirmed and tested in PCR with reference strains, previously identified by multisequence analysis of *calmodulin*,β*-tubulin, histone H3, translation elongation factor and internal transcribed spacer regions* and available in NCBI database (Moumni et al., 2019, 2020): *S. cucurbitacearum* (isolates D33, D49, D12, D83, ID1, and ID3), *Phoma* sp. (isolate Ph39), *Alternaria alternata* (isolates A38, A15), *Fusarium solani* (isolate F174), *Curvularia specifera* (isolate B170), *Paramyrothecium roridum* (isolate M123), *Albifimbria verrucaria* (isolate M144), and *Stemphylium vesicarium* (isolate P164).

Preliminary conventional PCR tests were set up to define the optimal concentrations of MgCl_2_ (0.8, 1.0, 1.2, 1.5, and 2 mM), primers (0.5 and 1.0 μM), and template. The amplification cycling was: 95°C for 2 min, followed by 35 cycles of 95°C for 30 s, 55–60°C for 30 s, and 72°C for 30 s, and with a final extension cycle at 72°C for 7 min. The PCR products were visualized on 1.5% agarose gels in Tris-acetate buffer (40 mM Tris-acetate, 1 mM EDTA, and pH 8.0) after staining with GelRed (Biotium, United States), to confirm the specificity, expected size of the PCR product, and the sensitivity of the diagnostic system.

The specific amplicons were sequenced in both directions by Genewiz (Germany) according to Sanger Method. Nucleotide sequences were carefully checked reading the chromatogram in order to exclude ambiguous peaks, and then formatted in fasta format in order to carry out Megablast, optimized for highly similar sequences, in NCBI database nucleotide collection (nr/nt).

The total DNA extracted from *S. cucurbitacearum* strain D33 was diluted to different concentrations (285−2.9 × 10^–5^ ng). In addition to this determination of the right quantity of DNA for the amplification, serial dilutions were set up for seed sample T85 (285, 57, and 20 ng), as a seed sample that was highly contaminated by *S. cucurbitacearum* (65.4%) according to the blotter analysis.

### Molecular Survey of *Stagonosporopsis cucurbitacearum* on Naturally Infected Seeds by Conventional Polymerase Chain Reaction

The total DNA extracted from 19 of the seed samples with different *S. cucurbitacearum* infestations were processed through the conventional PCR using the DBF1/R1 primer pair at the optimized mix concentration and thermocycling conditions according to the preliminary tests. DNA of isolate D33 was used as the positive control. After the amplification, five specific amplicons were sequenced and compared with the database.

### Validation of *Stagonosporopsis cucurbitacearum* Identification by qPCR

The primer pair DBF1/R1 was tested in qPCR. The analytical sensitivity (limit of detection; LOD, Bustin et al., 2009) was assessed using serial dilutions (285 ng, 57 ng, 20 ng, 11.4 ng, 2.28 ng, 4.56 × 10^–1^ ng, 9.12 × 10^–2^ ng, 1.82 × 10^–2^ ng, 3.6 × 10^–3^ ng, 7.2 × 10^–4^ ng, 1.45 × 10^–4^ ng, and 2.9 × 10^–5^ ng) of DNA extracted from the mycelia of *S. cucurbitacearum* isolate D33 spiked with 20, 57, and 285 ng of DNA from healthy seeds in qPCR (Landi et al., 2021). The reactions were carried out in a total volume of 12 μl containing: 2× Master mix iTaq Universal SYBR Green Supermix (Bio-Rad Laboratories, Hercules, CA, United States), and 0.10 μM of the designed primers (DBF1/R1). The reactions were subjected to the following conditions: 3 min at 95°C, followed by 40 cycles of 20 s at 95°C, and 40 s at 60°C. The final step included the melting curve analyses from 65 to 95°C. Two technical replications were performed for three independent experiments (*n* = 6).

### Validation, Molecular Survey, and Absolute Quantification of *Stagonosporopsis cucurbitacearum* on Naturally Infected Seeds by Digital Droplet Polymerase Chain Reaction

The primer pair DBF1/R1, previously validated in conventional and qPCR, were tested in ddPCR with the same samples previous analyzed for qPCR validation. The ddPCR inhibitors, the optimal concentration of DNA template, the LOD and limit of quantification (LOQ) (Huggett et al., 2013) were determined. Here, 20 μl of the reaction mixture, containing 1 × QX200 ddPCR EvaGreen supermix (Bio-Rad), 150 or 300 nM each primer, and 20 ng of template, was transferred to a DG8 cartridge for droplet generation (QX200 droplet generator; Bio-Rad, Hercules, CA, United States). Then 70 μl droplet generation oil (Bio-Rad) was added to the cartridge, which was placed into the droplet generator. Droplet generation of 40 μl was carefully transferred to the ddPCR 96-well PCR plates (Bio-Rad), and the plates were sealed at 180°C using a PCR plate sealer (PX1; Bio-Rad).

The amplification was performed in a thermal cycler (ICycler; Bio-Rad), with a ramp rate of 2°C/s, with the following protocol: initial denaturation at 95°C for 5 min, then 45 cycles of denaturation at 95°C for 30 s, annealing at 60°C for 45 s (temperature ramp, 2°C/s), and finally, incubation at 98°C for 10 min, and storage at 4°C. After the cycling, the 96-well plates were fixed into a plate holder and positioned in the droplet reader (QX200; Bio-Rad). The droplets of each sample were analyzed sequentially, and the fluorescent signals of each droplet were measured individually by a detector. The droplets were read in the droplet reader, and then the ddPCR data were analyzed using Quantasoft version 1.7, which defined an automatic threshold or with a selected manually defined threshold applied. This incorporated the calculation of the basic parameters of the ddPCR (e.g., concentrations, mean amplitudes of positive, and negative droplets), and the mean copies per partition and total volume of the partitions measured, as defined by the digital MIQE guidelines (Huggett et al., 2013). Two positive droplets were enough to determine a sample as positive, and only the reactions with more than 10,000 accepted droplets were used for analysis.

After a preliminary validation of the technique, the primers DB1F/R1 were used at the concentration of 150 nM and 20 ng of total DNA extracted from 13 of the seed samples, which were representative of the different levels of pathogen contamination (as previously defined with the blotter analysis and conventional PCR) were processed by ddPCR (QX200 system; Bio-Rad, Hercules, CA, United States), according to the manufacturer instructions.

## Results

### Incidence of *Stagonosporopsis cucurbitacearum* Using the Blotter Method

The level of squash seed infection detected for *S. cucurbitacearum* using the blotter test ranged from 0 to 65.4% (Table 1). *S. cucurbitacearum* was detected for 29 of the 31 seed samples collected (93.6% of samples). More than 20% incidence of seedborne *S. cucurbitacearum* was detected for 12 of these seed samples (Table 1).

**TABLE 1:** Incidence of *Stagonosporopsis cucurbitacearum* infection using the blotter method for the 31 squash seed samples collected in 2015, 2016, and 2017.

Year	Sample code	*S. cucurbitacearum* incidence on seeds (%)^a^	Selected for further analysis
**2015**	T4	44.0 ± 4.5 abcd	Yes
	T5	4.2 ± 2.2 fg	–
	T7	57.0 ± 4.1 ab	Yes
	T8	21.5 ± 4.5 def	Yes
	T9	62.0 ± 4.2 a	Yes
	T11	29.2 ± 5.7 bcdef	Yes
	T12	4.4 ± 1.9 fg	Yes
	T13	4.0 ± 4.0 fg	–
	T14	4.5 ± 1.5 fg	–
	T18	0.0 ± 0.0 g	–
	T19	9.0 ± 2.0 fg	–
	T22	3.5 ± 1.7 fg	–
	T82	50.0 ± 4.1 ab	Yes
**2016**	T83	49.0 ± 4.1 abc	Yes
	T84	26.5 ± 3.6 cde	Yes
	T85	65.4 ± 5.2 a	Yes
	T86	25.0 ± 2.3 d	Yes
	T87	11.0 ± 2.2 ef	Yes
	T89	2.0 ± 0.9 g	–
	T90	1.5 ± 1.0 g	Yes
	T91	10.0 ± 2.7 efg	–
	T92	63.0 ± 4.5 a	Yes
	T93	6.1 ± 2.4 fg	Yes
	T94	9.0 ± 2.6 fg	–
	T95	15.0 ± 2.6 ef	Yes
**2017**	T96	11.0 ± 2.2 ef	Yes
	T97	1.5 ± 0.8 g	–
	T98	1.5 ± 0.8 g	–
	T99	8.0 ± 2.2 fg	–
	T100	26.0 ± 4.3 cdef	Yes
	T101	0.0 ± 0.0 g	Yes

*^a^For each sample, two replicates of 100 seeds were tested using the blotter analysis. Means followed by different letters indicate significant deviation based on Welch’s ANOVA and post hoc means separation using the Games-Howell test (P < 0.05).*

The molecular approach allowed to corroborate the data emerging during the morphological characterization, and speed up the process of identification. By a multiplex PCR able to distinguish the three morphologically similar species, the natural inoculum of seeds in our study was represented only by *S. cucurbitacearum* (data not shown).

### Molecular Detection of *Stagonosporopsis cucurbitacearum* by Conventional and Quantitative Real-Time Polymerase Chain Reaction

#### Optimization of Amplification Conditions and Primer Specificity by Conventional Polymerase Chain Reaction

The preliminary tests defined the optimal analysis conditions as 20 ng total DNA, 200 μM dNTPs mixture, 0.5 μM each primer, 1.2 mM MgCl_2_, 1.25 U Taq polymerase (Promega Corporation, Madison, WI, United States), and 20 ng template, in a total reaction volume of 25 μl. We set up the following cycling conditions: 95°C for 3 min., 35 cycles with 95°C for 30 s, 58°C for 30 s, and 72°C for 30 min, followed by a final extension step at 72°C for 5 min. The specificity tests amplified a 208-bp specific fragment in isolates D33, D12, D49, ID1, and ID3, which had been previously identified as *S. cucurbitacearum* by multilocus sequence analysis (Moumni et al., 2019, 2020; Supplementary Figure 1). The fragment was sequenced and analyzed by BlastN. All of the fragments shared high nucleotide similarity (>99%) with the sequences available in the database.

No amplification was recorded for the water control and for the other fungal pathogen samples [*Phoma* sp. (Ph39), *Alternaria alternata* (A38, A15), *F. solani* (F174), *C. specifera* (B170), *P. roridum* (M123), *Albifimbria verrucaria* (M144), and *S. vesicarium* (P164)], which are commonly isolated from squash seeds (Moumni et al., 2020; Supplementary Figure 1).

#### Limit of Detection for Conventional Polymerase Chain Reaction

The sensitivity of the DBF1/R1 primers was evaluated using serial dilution of DNA extracted from *S. cucurbitacearum* mycelia. The minimum concentration of target DNA that could be detected with these primers was 9.12 × 10^–2^ ng (Supplementary Figure 2). An ambiguous amplification was recorded at 1.82 × 10^–2^ ng. The PCR amplification fragments were strongly visualized when the DNA concentrate of *S. cucurbitacearum* ranged from 40 to 10 ng, and weakly visualized with 1 ng template. Very low DNA concentration (<1.82 × 10^–2^ ng) were not amplified by the primers (Supplementary Figure 2).

**FIGURE 1:**
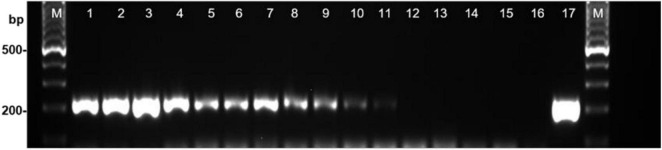
Detection of *Stagonosporopsis cucurbitacearum* from squash seed samples with different levels of infestation using PCR with primer pair DBF1/DBR1. Lanes 1–12: decreasing incidence of contamination (see Table 1): T85, T92, T9, T4, T86, T95, T96, T87, T93, T12, T90 and T101 (local healthy seed). Lane 13, commercial healthy seed (IHS); lane 14, water control (WC); lane 15, *Curvularia spicifera* (isolate B170); lane 16, *Phoma* sp. (isolate Ph39); lane 17, *S*. *cucurbitacearum* (isolate D33). M: Ladder, 100-bp.

Once the specificity and sensitivity of the primers for *S. cucurbitacearum* were established, the 19 seed samples were analyzed. In 17 of these 19, a specific fragment of ∼208 bp was detected using the DBF1/R1 primer pair, as for the reference strain D33 of *S. cucurbitacearum* (positive control). These primers amplified the DNA from the seeds naturally contaminated by *S. cucurbitacearum* down to a threshold of 4.5% incidence in 200 seeds, as indicated in the blotter analysis. Faint band was obtained when the seed samples were contaminated at ∼1.5% in the blotter analysis (Figure 1). The PCR primers consistently showed strong bands for the seed samples with incidence from 44.0 to 65.4%, moderate bands between 21.5 and 29.1%, weak bands between 4.2 and 4.5%, and a very faint band with 1.5% infection (Figure 1). No amplification was detected in samples T18 and T101, as also for the water control and the certified healthy seed sample (IHS).

The amplicons obtained from the DNA extracted from the mycelia (ID3 and ID9) and seed samples (T4and T7) were sequenced, and have been deposited with the NCBI database as MZ218113–MZ218116.

#### Validation of *Stagonosporopsis cucurbitacearum* Identification by qPCR

The reliable detection of DNA, extracted from *S. cucurbitacearum* isolate D33, ranged from 57 ng/reaction (Cq 22.01 ± 0.16) to 0.0182 ng/reaction (Cq 33.91 ± 0.8) (Table 2). The spiked sampled obtained with 20 ng/reaction of healthy seed DNA showed consistent results. For these concentrations, the Cq values was correlated with those obtained during the analysis of the DNA extracted from the mycelia of *S. cucurbitacearum* (Table 2). A single peak in positive samples suggests a single size product, with the melting temperature (TM) of 85.5°C (Supplementary Figure 3). At these conditions, it is reasonable to indicate as LOD in qPCR at Cq ≤ 33 (Table 2). Not consistent results were observed for *S. cucurbitacearum* mycelia DNA spiked with 57 and 285 ng of DNA from healthy seed.

**TABLE 2:** The *Stagonosporopsis cucurbitacearum* limits of detection estimated by qPCR method.

Fungal DNA (ng/reaction)	Healthy seed DNA qPCR (Cq mean ± SD)			
	0 ng	20 ng	57	285
285	Na	Na	Na	Na
57	22.01 ± 0.16	22.03 ± 0.07	22.83 ± 0.87	Na
20	23.53 ± 0.23	23.51 ± 0.14	23.13 ± 0.39	27.1 ± 2.2*
11.4	23.89 ± 0.40	24.19 ± 0.01	24.10 ± 1.01*	Na
2.28	26.29 ± 0.26	26.51 ± 0.20	26.50 ± 0.70	Na
0.456	29.28 ± 0.01	28.99 ± 0.26	28.39 ± 0.81	Na
0.0912	31.61 ± 0.1	31.56 ± 0.31	31.04 ± 1.21*	Na
0.0182	33.91 ± 0.8	33.7 ± 1.27	32.67 ± 1.02*	Na
0.0036	Na	Na	Na	Na
0.00072	Na	Na	Na	Na
0.000145	Na	Na	Na	Na

DNA of healthy seed (0, 20, 57, and 285 ng/qPCR reaction) spiked with serial dilutions of fungal DNA. The experiments were assessed in duplicate over three independent experiments (n = 6). Cq, quantification cycle; SD, standard deviation; na, not amplified, at these dilutions the probability of replicates detection of last dilution was absent or lower than 50%; *, two replicate amplified of six performed.

### Detection of *Stagonosporopsis cucurbitacearum* by Droplet Digital PCR

**FIGURE 2:**
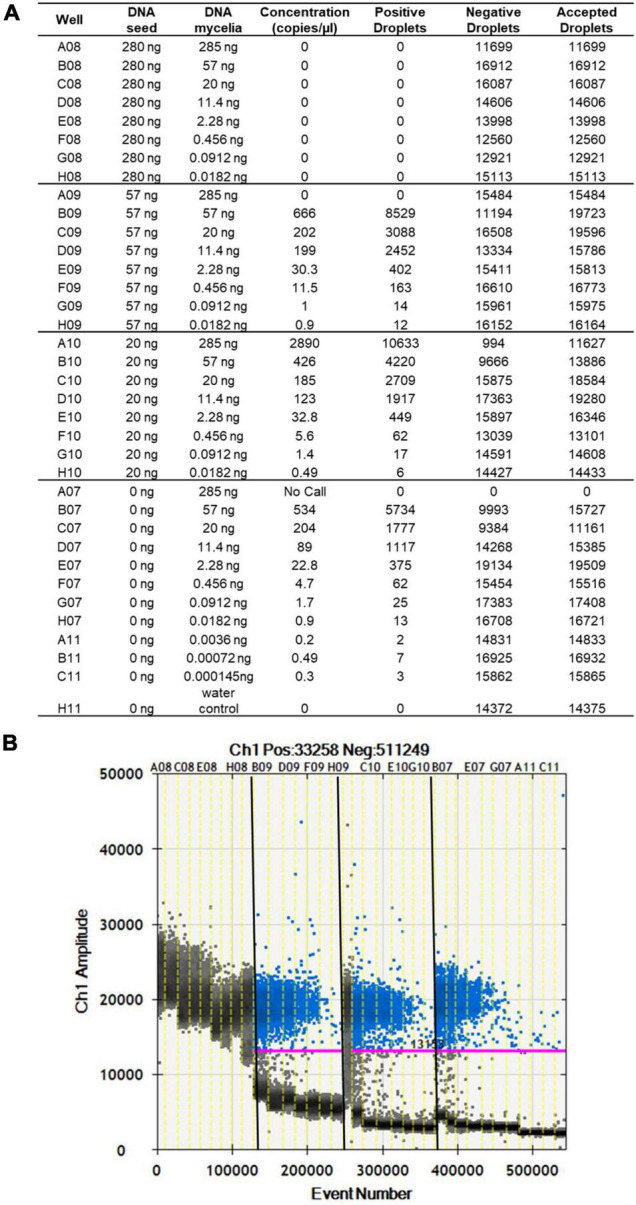
Analysis of spike samples obtained without and with mixing DNA from healthy seeds at different concentrations (as indicated) with DNA mycelia of *S. cucurbitacearum* at different concentrations (as indicated). **(A)** Absolute quantification. **(B)** Fluorescence amplitude. Pink line, threshold above which positive droplets (blue) contain at least one copy of target DNA, and below which negative droplets (gray) contain no copies of target DNA. Different sample sets in **(A)** are divided by vertical dotted yellow lines in **(B)**. Wells A08 to H08 showed saturation signals.

The limit of detection of *S. cucurbitacearum* by ddPCR was 3.6 × 10^–3^ ng, below which that there was no linear quantification of *S. cucurbitacearum* (Figures 2A,B). From the spiked samples analysis, a concentration of DNA > 20 ng had a negative influence on the detection of *S. cucurbitacearum* and on the accuracy of the absolute quantification (Figures 2A,B). The optimum condition was seen for 20 ng DNA from healthy seeds, at which the quantification of *S. cucurbitacearum* was highly related to the concentration of *S. cucurbitacearum* DNA added.

**FIGURE 3:**
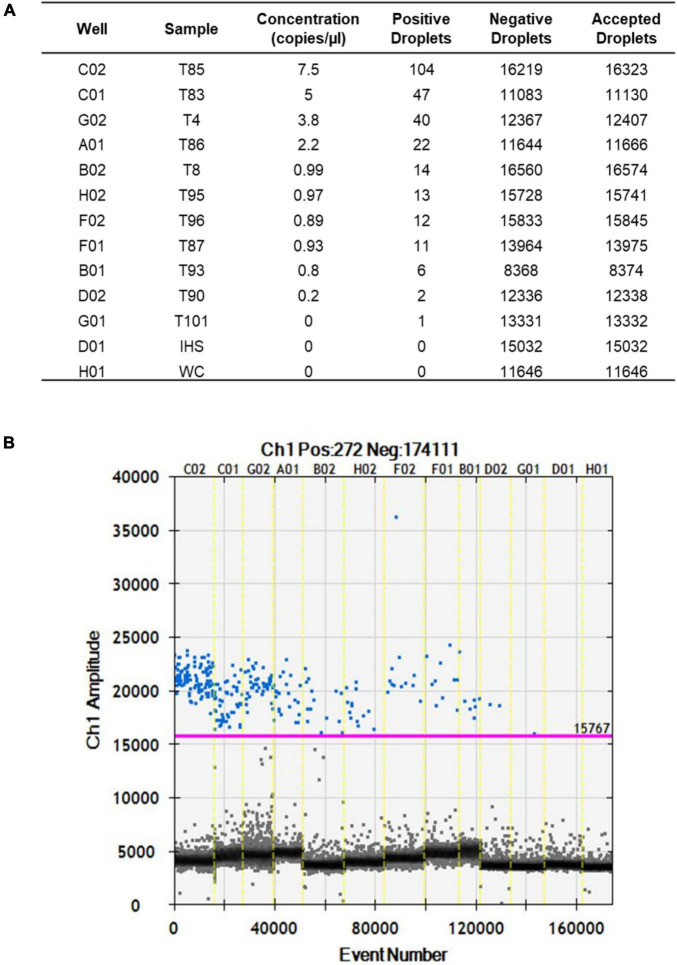
Fluorescence amplitudes of *S. cucurbitacearum* in the naturally infected seed samples (as indicated) and controls (IHS and WC) using ddPCR. **(A)** Absolute quantification. **(B)** Fluorescence amplitude.

After optimization of the amplification conditions, the ddPCR was applied to 13 seed samples that were naturally infected with *S. cucurbitacearum* and had been analyzed previously using the blotter analysis and the conventional PCR. The number of total events corresponding to the amount of droplets generated by the ddPCR ranged from 8,375 (sample T93) to 16,574 (sample T8) (Figures 3A,B). These values were related to the number of events, and ranged from 7.5 copies/μl (sample T85) to 0.2 copies/μl (sample T90). For sample T101, the water control and the healthy seeds sample (IHS), no amplifications were recorded (Figures 3A,B).

### Comparisons Between the Blotter Analysis, Conventional Polymerase Chain Reaction, and Droplet Digital PCR

The data from the blotter analysis were analyzed in relation to the data obtained for the ddPCR (Table 2), with high correlation seen (*R*^2^ = 0.986, *p* ≤ 0.01). The concentration of the amplified DNA target expressed as copies/μl was in accordance with seed contamination, in terms of the proportions of seeds infected by *S. cucurbitacearum*, recorded in blotter analysis. For the water control and the healthy seeds sample (IHS), where no infection was detected in the blotter analysis, no amplicons or positive events were seen for either conventional PCR or ddPCR. In sample T90, which showed seed infection of 1.5% in the blotter analysis, and where conventional PCR showed a light, and ambiguous band, the ddPCR showed an absolute quantification of 0.2 copies/μl. Finally, sample T101 that was negative in the blotter analysis and conventional PCR showed only 1 positive event for ddPCR, so this was also considered as negative in the ddPCR, along with the water control and the healthy seeds sample (Table 3).

**TABLE 3:** Comparative detection of *Stagonosporopsis cucurbitacearum* in the naturally infected seed samples, according to the blotter analysis and conventional PCR and ddPCR.

Sample	Blotter	Molecular		
code	method (%)	detection Conventional PCR*^b^*	ddPCR	
			Positive events	Absolute quantification (copies/μl)
**T86**	25.0 ± 2.3	+	22	2.2
**T93**	6.1 ± 2.4	+	6	0.8
**T83**	49.0 ± 4.1	++	48	5
**T87**	11.0 ± 2.2	+	11	0.89
**T101**	0.0 ± 0.0	–	1	0
**T8**	21.5 ± 4.5	+	17	0.99
**T85**	65.4 ± 5.2	+++	104	0.95
**T90**	1.5 ± 1.0	+/–	2	0.2
**T96**	11.0 ± 2.2	+	12	0.93
**T4**	44.0 ± 4.5	++	3.8	0.35
**T95**	15.0 ± 2.6	+	13	0.97
**IHS^a^**	0.0 ± 0.0	–	0	0
**WC^a^**	–	–	0	0

*^a^IHS, healthy seed control;*

*^b^WC, water control. –, no amplification; +/–, very weak amplification, +, weak amplification; ++, moderate amplification; +++, strong amplification.*

## Discussion

Healthy seeds are the start of healthy plants, and this is an essential requirement to safeguard the productivity of crops. GSB is a widespread disease and leads to significant losses in yield and quality for cucurbit crops worldwide (Keinath, 2011; Li et al., 2015; Yao et al., 2016; Zhao et al., 2020). In addition, the use of grafted cucurbits further increases the risk of GSB development from seedborne inoculum. Indeed, GSB was observed for grafted watermelon in Tunisia, which caused severe yield losses (Boughalleb et al., 2007).

*Stagonosporopsis cucurbitacearum* was recovered most frequently from *Cucurbita* spp. (Rennberger and Keinath, 2018; Zhao et al., 2018), and for squash seed the only fungal pathogen related to GSB in Tunisia and Italy (Moumni et al., 2019, 2020). Many other studies have demonstrated that infected seeds are the primary inoculum for GSB (De Neergaard, 1989; Sudisha et al., 2006; Keinath, 2011). The use of highly sanitized quality seeds decreases the primary inoculum in the field (Ciampi-Guillardi et al., 2020). Therefore, the detection of seedborne fungal pathogens is an important aspect for disease management.

The blotter method was appropriate in this study for the detection of *S. cucurbitacearum* in these seed samples. However, a drawback of this conventional method is that the morphological identification requires mycological skills and is time consuming; also, fungal contaminants can often mask the development of a pathogen. Indeed, the morphological characterization of *S. cucurbitacearum*, as well *S. citrulli*, *S. cariacae* and *Phoma* spp. are similar, and so distinguishing between these can be difficult (Keinath et al., 1995; Rennberger and Keinath, 2018). For this reason, in our study after the blotter tests and morphological identification, a preliminary molecular analysis carried out on the *S. cucurbitacearum*, as proposed by Brewer et al. (2015), allowed to confirm the identity. Rapid and accurate detection of pathogens transmitted by seeds should improve integrated disease management strategies, to control and prevent the spread of diseases caused by these pathogens. Several molecular methods have now been reported to detect pathogens on seeds (Lee et al., 2001; Pryor and Gilbertson, 2001; Samac et al., 1998). To set up efficient substitutes for the more traditional techniques, these methods need to be specific, sensitive, rapid, and adaptable to routine analysis. In the present study, the DBF1/DBR1 primers were designed to detect *S. cucurbitacearum* in these squash seeds. This set of primers successfully amplified the predicated size of the DNA fragment in infected material. One specific advantage of this PCR detection protocol is that it requires 1 day for completion, compared to the 10 days required for the blotter method. Thus, it can be used to examine both greater numbers and larger sample sizes with high reliability. Indeed, several studies have already reported primer pairs for the identification of *S. cucurbitacearum* in plant fragments after isolation to purity (Somai et al., 2002; Keinath et al., 2003; Brewer et al., 2015). Our analysis showed that the ddPCR method had a higher sensitivity than qPCR, and it is more reliable for the detection of the pathogen even at lowest titer.

Droplet digital PCR represents an innovative application in the diagnostic field, which is a user-friendly quantification technology, which does not require standard curve for the calibration (Bustin et al., 2009) that can be broadly used in several scientific fields, and its application to plant pathology is growing (Zhao et al., 2016). The present study represents the first approach to assess ddPCR as a reliable tool to detect and quantify pathogenic fungi associated with seeds. Many studies have reported that ddPCR is beneficial in terms of improved sensitivity of pathogen detection, and reduced effects of PCR inhibitors on PCR efficiency (Rački et al., 2014; Dupas et al., 2019; Bae et al., 2020).

The present study was thus designed to assess the diagnostic potential and sensitivity of ddPCR for absolute quantification of *S. cucurbitacearum* in the seeds of squash, as also compared to conventional PCR. ddPCR and the blotter test showed a high degree of correlation (*R*^2^ = 0.986, *p* ≤ 0.01) here. del Pilar Martínez-Diz et al. (2020) showed that ddPCR was more sensitive than qPCR for detection and quantification of the fungus *Ilyonectria liriodendri*. Dupas et al. (2019) demonstrated that ddPCR can improve the detection of the bacterium *Xylella fastidiosa* at low levels of infection and identified positive samples in those defined as negative by real-time PCR. Liu et al. (2020) suggested the use of ddPCR for detection of the fungus *Tilletia controversa* in soil samples and demonstrated that ddPCR was 100 times more sensitive than conventional PCR. Similarly, the data in the present study show that this ddPCR assay is a reliable alternative for quantification of *S. cucurbitacearum* on squash seeds. At the moment, for several important seedborne pathogens of vegetable crops, included *S. cucurbitacearum*, the International Seed Health Initiative and the American Seed Trade Association are attempting to compile pragmatic minimum thresholds based on experimental data, as well as empirical evidence and experience, that can be applied to seed in commerce. Considering that *S. cucurbitacearum* is transmitted by seeds and even low seed infection can cause medium-high economic losses in the field (Keinath, 2011), in our study we applied on one hand the blot test, an official method suggested by ISTA, on the other hand we explore the application of an absolute quantification method (ddPCR). To the best of our knowledge, this study is the first report of ddPCR for detection of such seedborne fungi. Further studies are required to evaluate and validate this new technology for routine use in the diagnosis of this and other seedborne pathogens. In addition, knowledge improvement of pathogen epidemiology initiated from seeds could lead to improve the management of such disease.

## Data Availability Statement

The datasets presented in this study can be found in online repositories. The names of the repository and accession number(s) can be found below: https://www.ncbi.nlm.nih.gov/; MZ218113–MZ218116.

## Author Contributions

SM, LL, and GR: conceptualization. SM, VM, and MM: data curation and investigation. SM, MM, LL, and VM: formal analysis. SM and LL: methodology. SM and GR: project administration. SM, GR, and MA: supervision. MM and SM: writing—original draft. SM, MM, LL, MA, and GR: writing—review and editing. All authors have read and agreed to the published version of the manuscript.

## Conflict of Interest

The authors declare that the research was conducted in the absence of any commercial or financial relationships that could be construed as a potential conflict of interest.

## Publisher’s Note

All claims expressed in this article are solely those of the authors and do not necessarily represent those of their affiliated organizations, or those of the publisher, the editors and the reviewers. Any product that may be evaluated in this article, or claim that may be made by its manufacturer, is not guaranteed or endorsed by the publisher.
