# Supplementary material for: Detection and Quantification of Stagonosporopsis cucurbitacearum in Seeds of Cucurbita maxima Using Droplet Digital Polymerase Chain Reaction
Source: Front Microbiol. 2022 Jan 11;12:764447. doi: 10.3389/fmicb.2021.764447 (PMC8788924; doi:10.3389/fmicb.2021.764447)
Supplement: Supplementary file 1 [file Data_Sheet_1.docx]

**Supplementary Tables and Figures**

**Supplementary Table S1.** Comparative detection of *Stagonosporopsis cucurbitacearum* in the naturally infected seed samples, according to the blotter analysis and conventional PCR and ddPCR.

| Code samples | Blotter method (%) | Molecular detection | | | |
| --- | --- | --- | --- | --- | --- |
|  |  | **Conventional**  **PCR^b^** | **ddPCR** | | |
|  |  |  | **Positive events** | | **Absolute quantification (copies/µl)** |
| T86 | 25 | + | 22 | 2.2 | |
| T93 | 6.1 | + | 6 | | 0.8 |
| T83 | 49 | ++ | 48 | | 5 |
| T87 | 11 | + | 11 | | 0.89 |
| T101 | 0 | - | 1 | | 0 |
| T8 | 21.5 | + | 17 | | 0.99 |
| T85 | 65.4 | +++ | 104 | | 0.95 |
| T90 | 1.5 | +/- | 2 | | 0.2 |
| T96 | 11 | + | 12 | | 0.93 |
| T4 | 44 | ++ | 3.8 | | 0.35 |
| T95 | 15 | + | 13 | | 0.97 |
| IHS^a^ | 0 | - | 0 | | 0 |
| WC^a^ |  | - | 0 | | 0 |

IHS, Healthy seed control; WC, water control

-, no amplification; +/-, very weak amplification, +, weak amplification; ++, moderate amplification; +++, strong amplification


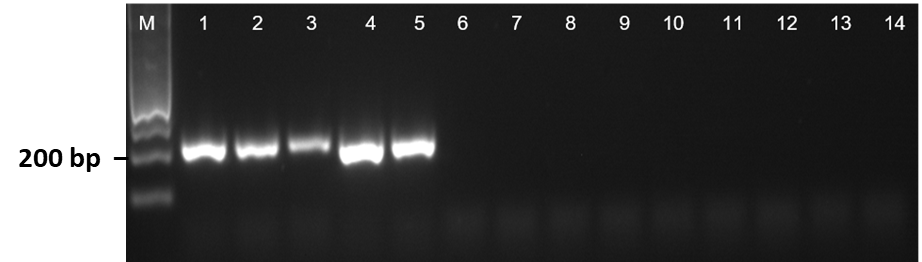


**Supplementary Figure S1.** Preliminary specificity test for the primer pair DBF1/R1. M, Ladder 100 bp. Lanes: 1-5, *Stagonosporopsis cucurbitacearum*; 6, *Phoma* sp*.;* 7, *Alternaria alternata*; 8, *A. Alternata*; 9, *Fusarium solani*; 10, *Curvularia specifera*; 11, *Paramyrothecium roridum*; 12, *Albifimbria verrucaria*; 13, *Stemphylium vesicarium;* 14, water control.


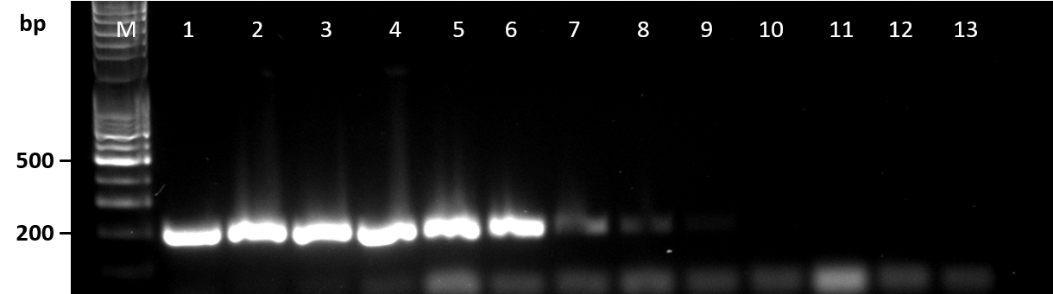


**Supplementary Figure S2.** Detection limit for primer pair DBF1/DBR1 specific for *Stagonosporopsis cucurbitacearum.* Primer pair was used in the PCR with serial dilutions of DNA extracted from *S. cucurbitacearum* mycelia (isolate D33). Lanes 1-13: 285, 57, 20, 11.4, 2.28 ng; 456, 91.2, 18.2, 3.6 pg; 720, 145, 29 fg. Lane 13, water control. M: molecular weight markers (100 bp).


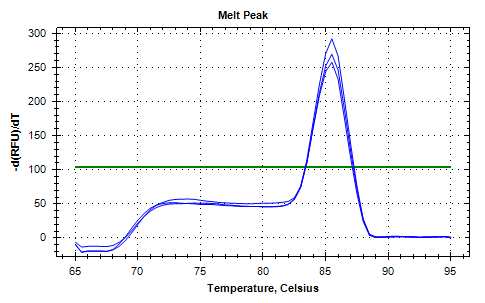


**Supplementary Figure S3.** The melting curve analysis of real-time qPCR obtained with primers DBF1/DBR1 for the detection of *Stagonosporopsis cucurbitacearum*. The figure shows a single peak at 85.5°C, related to melting temperature (Tm) of qPCR products using SYBR Green I.
